# Influence of Two Widely Used Solvents, Ethanol and Dimethyl Sulfoxide, on Human Sperm Parameters

**DOI:** 10.3390/ijms24010505

**Published:** 2022-12-28

**Authors:** Marie Bisconti, Philippe Grosjean, Vanessa Arcolia, Jean-François Simon, Elise Hennebert

**Affiliations:** 1 Laboratory of Cell Biology, Research Institute for Biosciences, Research Institute for Health Sciences and Technology, University of Mons, Place du Parc 23, 7000 Mons, Belgium; 2 Numerical Ecology Unit, StatForU, University of Mons, Place du Parc 23, 7000 Mons, Belgium; 3 Clinique de Fertilité Régionale de Mons, CHU Ambroise Paré Hospital, Boulevard Kennedy 2, 7000 Mons, Belgium

**Keywords:** human spermatozoa, capacitation, ethanol, dimethyl sulfoxide, sperm parameters, solvent

## Abstract

To study mechanisms involved in fertility, many experimental assays are conducted by incubating spermatozoa in the presence of molecules dissolved in solvents such as ethanol (EtOH) or dimethyl sulfoxide (DMSO). Although a vehicle control group is usually included in such studies, it does not allow to evaluate the intrinsic effect of the solvent on sperm parameters and its potential influence on the outcome of the experiment. In the present study, we incubated human spermatozoa for 4 h in a capacitation medium in the absence or the presence of different concentrations of EtOH and DMSO (0.1, 0.5, 1.0, and 2.0%) to assess the impact of these solvents on sperm motility, vitality, capacitation, and acrosome integrity. The presence of statistically significant relationships between increasing solvent concentrations and the investigated parameters was assessed using linear mixed models. A significant effect was observed with both solvents for total and progressive sperm motilities. We also evaluated the effect of time for these parameters and showed that the influence of the solvents was stable between 0 and 4 h, indicating an almost direct impact of the solvents. While EtOH did not influence sperm vitality and acrosome integrity, a significant effect of increasing DMSO concentrations was observed for these parameters. Finally, regarding capacitation, measured via phosphotyrosine content, although a dose-dependent effect was observed with both solvents, the statistical analysis did not allow to precisely evaluate the intensity of the effect. Based on the results obtained in the present study, and the corresponding linear mixed models, we calculated the concentration of both solvents which would result in a 5% decline in sperm parameters. For EtOH, these concentrations are 0.9, 0.7, and 0.3% for total motility, progressive motility, and capacitation, respectively, while for DMSO they are 1.5, 1.1, >2, 0.3 and >2% for total motility, progressive motility, vitality, capacitation, and acrosome integrity, respectively. We recommend using solvent concentrations below these values to dissolve molecules used to study sperm function in vitro, to limit side effects.

## 1. Introduction

The decline in male fertility observed worldwide over the past 80 years has led to a considerable increase in studies aimed at improving the diagnosis, treatment, and preservation of male reproductive health, as evidenced by the growing number of scientific publications [1]. At the same time, in recent years there has been a surge in the development of male contraceptives to free women from this constraint and to allow men greater reproductive autonomy [2,3]. Many studies are based on experimental tests involving the in vitro incubation of spermatozoa in the presence of molecules (or drugs) of interest. Most of these test molecules are poorly soluble in water, saline buffers, or culture medium and must be dissolved in organic solvents before being added to sperm. Dimethyl sulfoxide (DMSO) and ethanol (EtOH) are the most widely used solvents in sperm studies. They are used to dissolve specific molecules/chemical compounds to investigate various signaling pathways (e.g., [4,5,6,7,8,9,10,11]), fertilization mechanisms (e.g., [12,13,14]), the effect of environmental pollutants (e.g., [15,16,17,18,19,20,21,22]), antioxidant formulations (e.g., [23,24,25]), or novel male contraceptives (e.g., [26,27,28,29]). These solvents are also used to dissolve molecules required for the investigation of sperm parameters, such as acrosome reaction [30,31] or mitochondrial function [32]. In addition, DMSO is also used for the cryopreservation of sperm [33,34,35].

In somatic cells, it has been demonstrated that in vitro incubation with DMSO and EtOH induces important changes in cellular processes (e.g., [36,37,38,39,40,41]). Although results vary greatly according to the parameter studied and the cell line, it appears that very low concentrations of both solvents can already have an effect (e.g., 10^−9^% EtOH [37], 0.1% DMSO [41]). However, in spermatozoa, this effect has been poorly studied. This is particularly true for DMSO, although it is the most common vehicle in sperm studies.

Most of the studies aimed at investigating the effect of DMSO on sperm function were performed in the context of cryopreservation, to evaluate its potency as a cryoprotectant alone or in combination with semen extenders (i.e., soybean lecithin, skim milk, etc.). The observed response can be totally different according to the species studied, the experimental conditions, and the extender used. However, many studies showed that the use of DMSO as a cryoprotectant has a negative impact on sperm parameters, such as motility, vitality, and membrane and acrosome integrity in the post-thaw sperm in comparison to glycerol, the most commonly used cryoprotectant (e.g., [42,43,44,45,46,47,48,49]). To the best of our knowledge, no study aimed at investigating the global effects of DMSO alone (i.e., not combined with a semen extender for cryopreservation) on sperm function. However, sporadic information can be found for some studies in which the effect of DMSO was tested for the specific investigated experimental conditions (e.g., [6,8,28,29,50,51]). For instance, in humans, Gruber et al. [29] tested the influence of incubation of 10 min with increasing concentrations of DMSO on sperm curvilinear velocity (VCL), the parameter they considered in their study. An effect was only observed from 5% DMSO and the authors concluded that the use of 0.1% DMSO was safe to dilute the tested molecules for their screening assay.

In contrast to DMSO, more studies are found on the effect of EtOH on sperm function, mainly driven by the need to investigate the effect of excessive alcohol consumption on male fertility. The negative influence of increasing concentrations of EtOH (from 0 to 0.4%) in the sperm capacitation medium on sperm fertilizing ability has been demonstrated in humans [52,53], mice [54], hamsters [53], and bulls [55]. Results are less homogeneous regarding the influence of EtOH on sperm motility. Indeed, some studies showed no effect in mice (EtOH tested concentrations ranging from 0 to 0.4% [54]) and humans (0–0.5% [52], 0–1.15% [56]), while others reported a negative effect in hamsters (0–0.4% [53]) and humans (0.3 and 0.5% [57]). Moreover, the addition of EtOH in the sperm incubation medium was shown to decrease the number of spermatozoa with normal morphology in humans [57] and the level of the acrosome reaction in hamsters [53], and to induce the loss of acrosomes in humans [56].

The studies cited above demonstrate that both EtOH and DMSO can have a negative impact on sperm parameters, which are themselves recorded to study the effect of molecules of interest in many studies. However, data are scattered in the literature and the concentrations of EtOH and DMSO tested to measure their influence on sperm parameters are generally lower than the actual concentrations used to dissolve test molecules. Here, we investigated the impact of different concentrations of EtOH and DMSO on human sperm motility, vitality, capacitation, and acrosome integrity. We tested concentrations between 0 and 2% as this range covers the concentrations typically used in studies in which spermatozoa are incubated in the presence of test molecules (e.g., [26] (1.5% EtOH), [50] (1% DMSO), [58] (1.5% DMSO), [23] (0.5% EtOH), [28] (1% DMSO), [20] (0.1% EtOH), [9] (1% DMSO)). We also measured the effect of incubation time on sperm motility. Based on the results obtained, we developed statistical models to calculate the decrease in sperm parameters according to EtOH or DMSO concentrations between 0 and 2%.

## 2. Results

### 2.1. Parameters of Sperm Samples Included in This Study

Nine samples were included in this study. Their parameters, information from the donors, and the experiments for which they were used are presented in Table 1. All the samples were in the reference values defined by the WHO 2021 guidelines [59] regarding semen volume, pH, sperm count, and sperm motility. Noteworthy, some of the samples had <4% normal forms but we considered this to have a limited effect as, for each tested condition, comparisons were made within the same sample.

### 2.2. Influence of EtOH and DMSO on Sperm Parameters

The relationship between increasing concentrations of EtOH and DMSO on different parameters of human spermatozoa after 4 h of incubation in a capacitation medium was studied and results are described in the following sections. Raw data obtained for all the parameters are available in Tables S1 and S2.

#### 2.2.1. Sperm Motility

**Figure 1:**
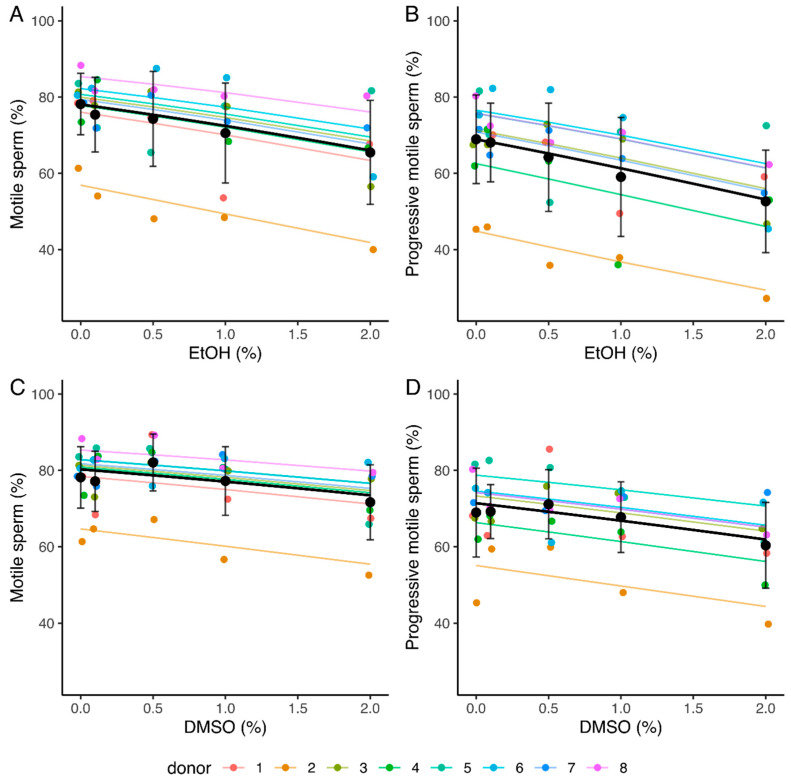
Influence of increasing concentrations of ethanol (EtOH) and dimethyl sulfoxide (DMSO) on total (**A**,**C**) and progressive (**B**,**D**) sperm motilities after a 4 h incubation. Values for each replicate (n = 8) are highlighted by a dot whose color refers to a donor. Colored lines represent the model fit for each donor. Black dots represent mean values for each concentration ± SD. Black lines correspond to the mean effect across all donors (Table 2 and Table S3).

A statistically significant reduction in both the total and progressive sperm motility values as EtOH concentration increases was observed (slopes of −0.30 and −0.34, with 95% confidence intervals [−0.39, −0.22] and [−0.41, −0.26] respectively) (Table 2 and Table S3, Figure 1A,B). The mean percentages of motile and progressive spermatozoa decreased from 78.2 and 69% (0% EtOH) to 65.5 and 52.6% (2% EtOH) respectively (Figure 1A,B). A significant decrease in total and progressive motility values as DMSO concentration increases was also observed (slopes of −0.19 [−0.28, −0.10] and −0.22 [−0.30, −0.13], respectively) (Table 2 and Table S3, Figure 1C,D). The mean percentage of motile and progressive spermatozoa decreased from 78.2% and 69% (0% DMSO) to 71.7% and 60.4% (2% DMSO), respectively (Figure 1C,D).

**Figure 2:**
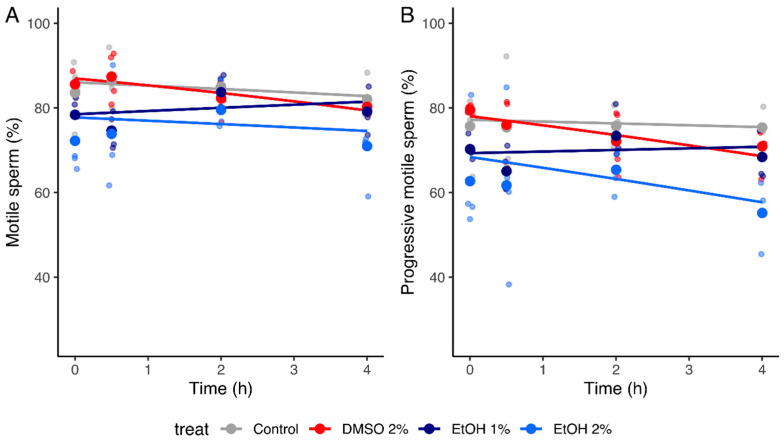
Influence of 1% and 2% ethanol (EtOH) as well as 2% dimethyl sulfoxide (DMSO) on total (**A**) and progressive (**B**) sperm motilities at various incubation times. Values for each replicate (n = 4) are highlighted by a small dot whose color refers to the tested condition (i.e., 1 or 2% EtOH or 2% DMSO). Large dots represent mean values for each tested incubation time within the same condition. Colored lines represent the mean model fit across all donors within the same condition. (Table 3 and Table S4).

To investigate the possibility of a time effect of the solvents, we recorded the sperm motilities in the absence or the presence of 1 and 2% EtOH and of 2% DMSO after different incubation times (0, 0.5, 2, and 4 h). For both concentrations of EtOH, values for total motility were significantly different at T = 0 h from those of control without EtOH (difference of intercept of −0.52 [−0.82, −0.25] and −0.57 [−0.85, −0.31] for 1 and 2% EtOH, respectively). Regarding DMSO, at T = 0 h, values for total motility did not differ from those measured in the control condition (difference of intercept of 0.079 [−0.20, 0.36] (Table S4, Figure 2). For the three tested conditions, no significant effect of time was observed by the generalized linear mixed models for the control and tested conditions (difference of slope of 0.11 [−0.0072, 0.23], 0.018 [−0.099, 0.14], and −0.075 [−0.19, 0.037] for 1% EtOH, 2% EtOH, and 2% DMSO, respectively (Table 3 and Table S4, Figure 2). However, it should be noted that the prediction power of the model is probably low due to the few data used (n = 4). Regarding progressive motility, it was not possible to develop a generalized linear model with the limited number of data available.

#### 2.2.2. Sperm Vitality

**Figure 3:**
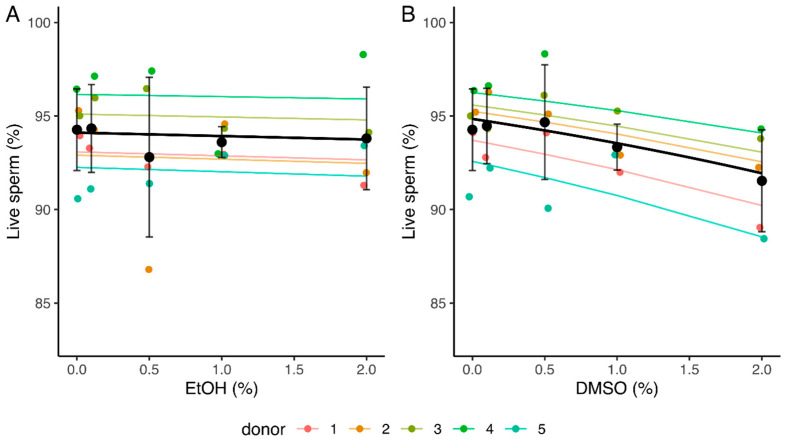
Influence of increasing concentrations of ethanol (EtOH) (**A**) and dimethyl sulfoxide (DMSO) (**B**) on sperm vitality after a 4 h incubation. Values for each replicate (*n* = 5) are highlighted by a dot whose color refers to a donor. Colored lines represent the model fit for each donor. Black dots represent mean values for each concentration ± SD. Black lines correspond to the mean effect across all donors (Table 2 and Table S3).

While EtOH did not seem to impact sperm vitality (slope of −0.032 [−0.16, 0.10]), a significant relationship was observed between the percentage of live spermatozoa and increasing concentrations of DMSO (slope of −0.24 [−0.37, −0.11]) (Table 2 and Table S3, Figure 3). The mean percentage of live spermatozoa decreased from 94.3% (0% DMSO) to 91.5% (2% DMSO) (Figure 3B).

#### 2.2.3. Sperm Capacitation

**Figure 4:**
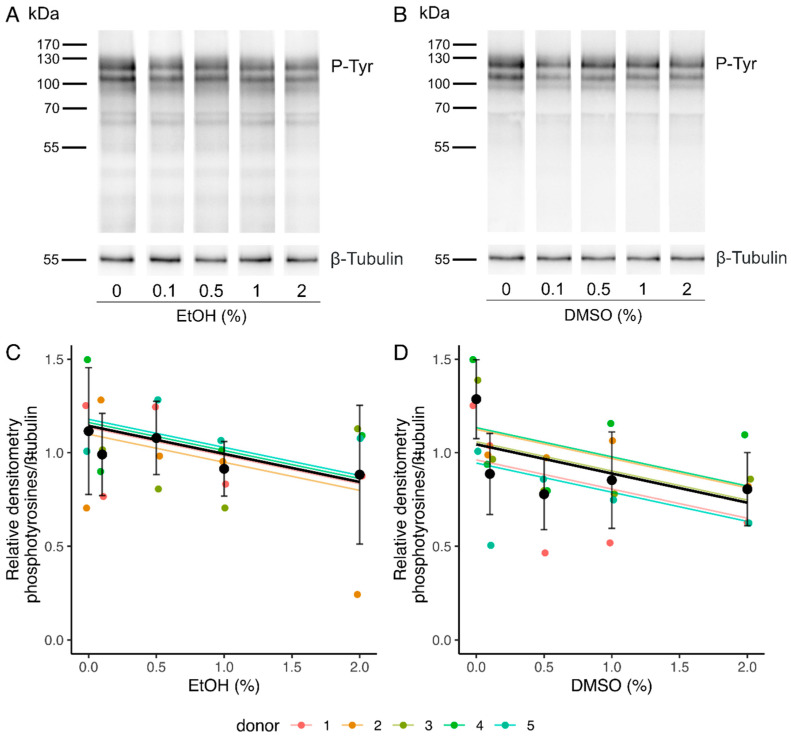
Influence of ethanol (EtOH) and dimethyl sulfoxide (DMSO) on sperm capacitation after a 4 h incubation. (**A**,**B**) Representative images of Western blot analyses of phosphotyrosilated proteins for spermatozoa incubated in the presence of EtOH (**A**) or DMSO (**B**). (**C**,**D**) Quantification of the level of phosphotyrosilated proteins in relative densitometry with respect to β-Tubulin for EtOH (**C**) and DMSO (**D**). Values for each replicate (n = 5) are highlighted by a dot whose color refers to a donor. Colored lines represent the model fit for each donor. Black dots represent mean values for each concentration ± SD. Black lines correspond to the mean effect across all donors (Table 2 and Table S3).

Sperm capacitation was assessed by phosphotyrosine analysis in Western blot, as tyrosine phosphorylation is recognized as a hallmark for sperm capacitation [60,61]. After a 4 h incubation in the capacitation medium, labeling was observed in all the samples at the level of three main bands comprised between 90 and 120 kDa (Figure 4A,B). The quantification of the level of phosphotyrosilated proteins was therefore performed using relative densitometry of these 3 bands with respect to β-Tubulin. A decrease in the intensity of the bands was observed by the linear mixed-effects model with increasing concentrations of EtOH and DMSO (slopes of −0.15 [−0.28, −0.0048] and −0.16 [−0.29, −0.021], respectively) (Table 2 and Table S3, Figure 4C,D).

#### 2.2.4. Acrosome Integrity

**Figure 5:**
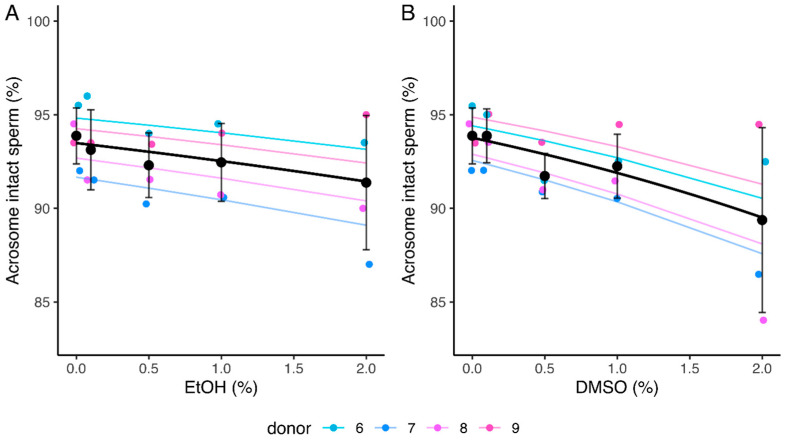
Influence of ethanol (EtOH) (**A**) and dimethyl sulfoxide (DMSO) (**B**) on sperm acrosome integrity after a 4 h incubation. Values for each replicate (*n* = 4) are highlighted by a dot whose color refers to a donor. Colored lines represent the model fit for each donor. Black dots represent mean values for each concentration ± SD. Black lines correspond to the mean effect across all donors (Table 2 and Table S3).

No significant relationship was observed between increasing concentrations of EtOH and the percentage of acrosome-intact spermatozoa (slope of −0.15 [−0.32, 0.022]). However, a significant decrease was observed for this parameter in the presence of DMSO (slope of −0.28 [−0.43, −0.12]) (Table 2 and Table S3, Figure 5). The mean percentage of acrosome-intact spermatozoa decreased from 93.9% (0% DMSO) to 89.4% (2% DMSO) (Figure 5B).

## 3. Discussion

Many studies related to male fertility involve the incubation of spermatozoa with test molecules to understand sperm function, to evaluate the effect of environmental pollutants, or discover new therapeutic strategies. Many of these molecules must be dissolved in organic solvents, which alone could influence the outcome of the experiments. In the present study, we evaluated the effect of different concentrations of EtOH and DMSO, the most common solvents used in sperm studies, on sperm motility, vitality, capacitation, and acrosome integrity. The results obtained allowed the development of linear mixed models to predict the impact of both solvents at concentrations between 0 and 2% for these parameters by means of parametric bootstrapped confidence intervals on the slope of the models (see https://github.com/STATforU/sz_etoh_dmso for all calculated predictions). This approach yields a reliable estimate of the solvent concentration effects without requiring strict homoscedasticity or normality of the residuals (such conditions are required for ANOVAs but they are near to impossible to assess adequately with a small number of replicates, *n* = 4–8 in our case).

Incubation of mature human spermatozoa for 4 h in a capacitation medium in the presence of increasing concentrations of EtOH and DMSO induced a significant decrease in both total and progressive motilities. Based on the generalized linear mixed models, we calculate that the maximum tested EtOH concentration (2%) would induce an 11% (with 95% confidence interval [8%, 15%]) decline in the percentage of motile spermatozoa and a 16% [12%, 19%] decline in the percentage of progressive spermatozoa in comparison to the medium without EtOH. Other studies showed an impact of sperm incubation with EtOH on sperm motility. In humans, a 5% decline in the percentage of progressive spermatozoa was recorded by Donnelly et al. [57] after a 4 h incubation of the spermatozoa in the presence of 0.5% EtOH. This value is close to the 4% [3%, 5%] decline predicted by our models using the same condition. In hamsters, a high decrease (21%) was observed for the number of motile spermatozoa after capacitation for 4 h in the presence of 0.4% EtOH [53]. This is much higher than the 2% [1%, 3%] decline we calculated for the same EtOH concentration using our linear mixed model. Oppositely, others reported no effect of sperm capacitation in the presence of EtOH on sperm motility in mice (tested concentrations ranging from 0 to 0.4% [54]), rats (1% [62]), and humans (0–0.5% [52], 0–1.15% [56]). These differences are likely due to differences in the experimental setup used (e.g., incubation medium, sperm preparation, sperm motility recording). DMSO appeared to have a lower effect on sperm motility than EtOH, with the 2% solution inducing a 7% [3%, 10%] decline in the percentage of motile spermatozoa and a 10% [6%, 14%] decline in the percentage of progressive spermatozoa. Some studies using DMSO as a solvent for the test molecule compared the impact of DMSO at concentrations between 0 and 1% to that of the medium without DMSO and showed no significant decrease in the motility of human [28,29,50,63,64], mouse [7], and rat [62] spermatozoa. These results are similar to those we obtained with the same concentrations (i.e., only a 4% [2%, 5%] decline with the 1% solution).

To investigate a potential time-dependent effect of EtOH and DMSO on sperm motility, we incubated spermatozoa in the presence of 1 and 2% of both solvents at different incubation times between 0 and 4 h. Due to the low amount of data, it was not possible to obtain a statistical model for progressive motility. The fitted linear mixed models showed that, at T = 0 h, the number of motile spermatozoa in the presence of 1 and 2% EtOH was significantly different from that of the control condition without EtOH, while no difference was measured with 2% DMSO. Moreover, no significant relationships were observed with the incubation time for the three conditions. For EtOH, these results imply that its influence on sperm motility would be almost direct (noteworthy, the T = 0 h in our experiments corresponds rather to T = 5 min because of the time required to take the videos of the spermatozoa). However, for DMSO, since no difference was observed with the control at T = 0 h and sperm motility was stable according to time, this would mean that there should be no difference at T = 4 h either. These results are contradictory to those measured in the experiment in which different concentrations of DMSO were tested during a 4 h incubation and for which a statistically significant dose effect was observed. As three of the four samples used to study the time effect were part of the eight samples used to investigate the dose effect, these contradictory results are presumably due to the low amount of data used to develop the linear mixed models.

Sperm vitality did not seem to be impacted in the presence of EtOH. These results are consistent with those from Alvarez et al. [56], who did not observe any change in the vitality of human spermatozoa after a 6 h incubation in the presence of 1.15% EtOH. However, a significant relationship was measured between the percentage of live spermatozoa and the concentration of DMSO, with a calculated 3% [1%, 6%] decline after a 4 h incubation in the presence of 2% DMSO. This effect, although statistically significant, is very low. These results are consistent with those obtained in other studies which compared the viability of human spermatozoa incubated in the presence of concentrations between 0 and 1% DMSO and showed no statistically significant difference with the medium without DMSO after a 3–4 h incubation [63,64,65].

We also investigated the impact of EtOH and DMSO on sperm phosphotyrosine content, as a marker of capacitation [60,61]. A significant effect of increasing EtOH and DMSO concentrations was observed. A decline of 30% [1%, 57%] and 31% [4%, 58%] in the phosphotyrosine content in the presence of 2% EtOH and 2% DMSO, respectively, were predicted by the linear mixed-effects models. However, the very large 95% confidence intervals indicate that more data are needed to develop a more precise model and draw accurate conclusions. Several studies on humans, hamsters, mice, and bulls showed a decrease in sperm fertilizing ability with increasing concentrations of EtOH (0–0.4%) [52,53,54,55]. As this effect was observed when EtOH was added in the capacitation medium used to incubate the spermatozoa prior to the fertilization tests but was not observed when EtOH was only added at the time of fertilization, the authors deduced that EtOH affects fertilization by inhibiting sperm capacitation. According to our linear mixed-effects models, a 0.4% EtOH concentration would induce a 6% [0%, 11%] decline in the phosphotyrosine content, indicating that capacitation could indeed be affected in these conditions.

Incubation of spermatozoa for 4 h in the capacitation medium in the presence of increasing concentrations of EtOH did not impact acrosome integrity. These results are in line with the lack of effect of 1% EtOH observed by Uguz et al. [62] during a 4 h incubation of rat spermatozoa under capacitating conditions. As for DMSO, a significant relationship between increasing concentrations and the percentage of acrosome-intact spermatozoa was observed. However, this effect is low, with the 2% solution predicted to induce a 4% [2%, 7%] decline in the percentage of spermatozoa with intact acrosome. These results are consistent with those reported by Naz [66], who showed no impact of a 6 h capacitation in the presence of 0.5% DMSO on the percentage of acrosome-less human spermatozoa in comparison to the capacitation medium containing phosphate-buffered saline (PBS) instead of DMSO. Similar results were obtained with boar (24 h incubation with 0.01% solution [67]), rat (4 h incubation with 1% solution [62]), and lama spermatozoa (4 h incubation, unknown concentration [68]).

Overall, sperm motility was the main parameter impacted by incubation in the presence of EtOH and DMSO. Although we did not investigate the molecular pathways involved in the impact of the solvents on sperm motility, the very quick effect observed with EtOH is reminiscent of an unspecific action on ion channels, such as Catsper, as shown for many drugs [69,70,71]. Indeed, Catsper regulates the intracellular calcium concentration and, thereby, sperm motility [72,73]. In addition, as sperm motility depends on the generation of ATP, one can hypothesize that oxidative phosphorylation and/or glycolysis pathways, the two metabolic pathways responsible for ATP production in spermatozoa [74], are impacted by the solvents. One way to verify this hypothesis would be to compare the ATP content in the spermatozoa incubated in the absence or presence of the solvents. While EtOH does not influence sperm vitality and acrosome integrity, a low effect of DMSO was measured for these parameters. Finally, regarding capacitation measured via phosphotyrosine content, although an impact was observed with both solvents, the developed model does not allow us to precisely evaluate the intensity of the effect.

## 4. Materials and Methods

### 4.1. Subjects and Ethics

Human semen samples were obtained from the fertility clinic of Ambroise Paré Hospital (Mons, Belgium) from patients undergoing routine semen analysis or from voluntary donors. All experiments conducted in this study were approved by the Ethics Committee of Ambroise Paré Hospital in Mons and by the Ethics Committee of Erasme Hospital in Brussels (protocol P2017/540) and the semen samples were obtained with informed written consent from all subjects. Semen was collected by masturbation after an abstinence period of three to five days and routine seminal analysis was performed according to the World Health Organization (WHO) 2021 guidelines [59]. Only samples whose sperm concentration and motility were within the reference values provided by the WHO 2021 guidelines were included in the study (see Table 1).

### 4.2. Sperm Preparation

Spermatozoa were purified from the semen samples by centrifugation at 300× *g* for 20 min at 37 °C on a discontinuous PureSperm 40/80 density gradient (Nidacon, Mölndal, Sweden) as described in [75] and the WHO guidelines [59]. Purified spermatozoa recovered from the bottom of the 80% PureSperm fraction were then washed at 600× *g* for 10 min at 37 °C with Dulbecco’s phosphate-buffered saline (DPBS). Spermatozoa were counted on a Makler Chamber and maintained at 37 °C until use.

For all the experiments, spermatozoa (2 × 10^6^ cells/mL) were incubated at 37 °C in an incubator containing 5% CO_2_ in a capacitation medium (HAM’s F-10 Nutrient Mix (Gibco, ThermoFisher Scientific,Waltham, MA, USA) containing 3 mg/mL HSA (Gynemed, Lensahn, Germany) and 100 µg/mL ampicillin) supplemented or not with EtOH (E/0665DF/17, ThermoFisher Scientific, Waltham, MA, USA) or DMSO (BP231, ThermoFisher Scientific, Waltham, MA, USA). In the first experiment, four concentrations of each solvent (0.1, 0.5, 1, and 2%) were tested during an incubation of 4 h. This incubation time was selected as it corresponds to that used in many studies in which sperm are incubated in the presence of test molecules. Moreover, it allows sperm capacitation (e.g., [76,77,78]), one of the parameters we investigated in the present study (see Section 4.5). At the end of the incubation, the influence of the solvents on different sperm parameters was investigated as described in the following sections. In a second experiment, different incubation times (0, 0.5, 2, and 4 h) were tested for some selected concentrations. In that case, only sperm motility was assessed.

### 4.3. Assessment of Sperm Motility

Motility analysis was performed by loading 2 µL of sperm suspension in 10 µm Leja counting chamber slides (SC 10-01-04-B, Microptic, Barcelona, Spain) maintained at 37 °C and 10–15 videos (5 s, 50 fps) corresponding to different fields of the chambers were recorded using a DFK 33UP1300 USB 3.0 color industrial camera connected to an inverted Eclipse Ts2R microscope (Nikon, Amstelveen, The Netherlands) with a 10× negative phase contrast objective. All spermatozoa in each video were analyzed with the Motility Module of the OpenCasa system v2.0 [79] to evaluate sperm total and progressive motilities. Results were checked manually to avoid counting the same sperm twice. Progressive spermatozoa were differentiated from non-progressive spermatozoa by eye as those swimming actively, either linearly or in a large circle. Where it was unclear whether sperm should be classified as motile or progressively motile, we applied a threshold VSL of 10 µm/s (as calculated by OpenCASA) (e.g., [80,81]). About 50 to 300 spermatozoa were analyzed per replicate for each condition.

### 4.4. Assessment of Sperm Vitality

A 10 μL aliquot of each sample was mixed with 30 μL of BrightVit solution (Microptic, Barcelona, Spain). After 5 min incubation at 37 °C, 25 μL was spread and dried on microscope slides, and mounted with Eukitt mounting medium (EUK-NEO-100, Microptic, Barcelona, Spain). The BrightVit solution is a hypo-osmotic medium that allows the swelling of living cells. The solution is composed of dyes including eosin that penetrates the membranes of dead cells, staining them pink, while living cells remain colorless. In this study, only the hypo-osmotic swelling test was used to determine sperm vitality and 300 spermatozoa were analyzed per replicate for each condition.

### 4.5. Assessment of Sperm Capacitation

Aliquots of the samples, containing 0.4 × 10^6^ spermatozoa, were centrifuged at 2000× *g* for 5 min at 4 °C. The spermatozoa were washed 3 times with cold phosphate-buffered saline (PBS, pH 7.4) and, after the last wash, the supernatant was removed and the spermatozoa were flash-frozen in liquid nitrogen and stored at −80 °C. Proteins were extracted in SDS sample buffer (50 mM Tris, 10% Glycerol, 2% SDS, 100 mM DTT), heated at 95 °C for 10 min, centrifuged, and loaded on 10% SDS-PAGE gels. After electrophoresis, the proteins were transferred onto PVDF membranes (GE Healthcare, Freiburg, Germany) using 25 mM Tris, 192 mM glycine, 0.05% SDS, and 20% methanol as transfer buffer. The membranes were washed with PBS containing 0.05% Tween 20 (PBS-T) and then blocked for 1 h at room temperature in the same buffer containing 5% BSA. The membranes were incubated overnight with anti-phosphotyrosine clone 4G10 monoclonal antibodies (05-321X, Merck, NY, USA) diluted 1:20,000 in PBS-T containing 3% BSA. After 5 washes of 5 min in PBS-T, HRP-conjugated Goat anti-mouse immunoglobulins (G21040, ThermoFisher Scientific, Waltham, MA, USA) diluted 1:50,000 in PBS-T containing 3% BSA was applied for 1 h. Finally, the membranes were washed again and immunoreactive bands were visualized using the ECL Western Blotting Substrate (32106, ThermoFisher Scientific, Waltham, MA, USA) and the Fusion FX imaging system (Vilber, Marne-la-Vallée, France).

### 4.6. Evaluation of Acrosome Integrity

Aliquots of spermatozoa (1 × 10^6^) contained in the capacitation solution were mixed in a 1:1 ratio with 4% paraformaldehyde (PFA) in PBS for 20 min at room temperature, for fixation. The samples were then centrifuged at 2000× *g* for 5 min. They were washed twice with 0.05 M glycine in PBS and once with PBS. Then, a total of 0.05 × 10^6^ spermatozoa were air-dried on a microscope slide and stained with a Coomassie solution (0.22% Coomassie Blue G-250, 50% methanol, 10% acetic acid, and 40% water) for 2 min as described in [82]. The slides were thoroughly rinsed with distilled water, air-dried, and mounted with a coverslip and Eukitt mounting medium (EUK-NEO-100, Microptic, Barcelona, Spain). This staining method allows to distinguish acrosome-intact spermatozoa from acrosome-less spermatozoa (Figure S1). A minimum of 200 spermatozoa was analyzed per replicate for each condition using an inverted Eclipse Ti2-U microscope (Nikon, Amstelveen, The Netherlands).

### 4.7. Statistical Analyses

Statistical analyses were performed using R 4.1.3 (R Foundation for Statistical Computing, Vienna, Austria). As the independent variable (i.e., the concentration of EtOH or DMSO) is quantitative and varies continuously, and considering the donors as random factors, linear mixed models were used to investigate the influence of EtOH and DMSO on sperm parameters.

For the analysis of sperm motilities, vitality, and acrosome integrity, each parameter of interest was separately fitted using a generalized linear mixed model with a logit link and assuming a binomial distribution to characterize how it varied in response to the concentration of solvent. The models were specified as:

$$X_{i}∼\mathrm{Binomial}\left(n=1,{\mathrm{prob}}_{X=1}=\hat{P}\right),$$

(1) $$\log\left[\frac{\hat{P}}{1-\hat{P}}\right]=\alpha_{j\left[i\right]}+\beta_{1}\;(\mathrm{concentration}),\;$$

*α*_*j*_ ∼ *N* (*μ*_*α*j_, *σ*^2^_*α*j_), for donor j = 1, …, J

where X corresponds to the studied parameter.

For capacitation (C), densitometry data for phosphotyrosine content were normalized to the loading control (β-Tubulin) and fitted with a linear mixed-effects model. The model was specified as:

C_*i*_ ∼ *N* (*α*_*j*[*i*]_ + *β*1(concentration), *σ*^2^) (2)

*α*_*j*_ ∼ *N* (*μ*_*α*j_, *σ*^2^_*α*j_), for donor j = 1, …, J

For the analysis of the motility over time (MT) in the presence of 1% and 2% EtOH as well as in the presence of 2% DMSO, data were fitted with a generalized linear model. The model was specified as:

$${\mathrm{MT}}_{i}∼\mathrm{Binomial}\left(n=1,{\mathrm{prob}}_{\mathrm{mot}=1}=\hat{P}\right)$$

(3) $$\begin{array}{cc} \log\left[\frac{P}{1-\hat{P}}\right]= & \alpha_{j\left[i\right]}+\beta_{1}\left({treat}_{DMSO\;2\%}\right)+\beta_{2}\left({treat}_{EtOH\;1\%}\right)\\ & +\beta_{3}\left({treat}_{EtoH\;2\%}\right)+\beta_{4}\left(\mathrm{time}\right)\\ & +\beta_{5}\left(\mathrm{time}\times {treat}_{DMSO\;2\%}\right)\\ & +\beta_{6}\left(\mathrm{time}\times {treat}_{EtOH\;1\%}\right)\\ & +\beta_{7}\left(\mathrm{time}\times {treat}_{EtOH\;2\%}\right) \end{array}$$

*α*_*j*_ ∼ *N* (*μ*_*α*j_, *σ*^2^_*α*j_), for donor j = 1, …, J

where treat corresponds to the treatment applied.

Non-singularity and convergence were checked by refitting the models with different optimizers. An analysis of Pearson’s residuals was performed for each model in the limits of the validity of such an analysis with generalized linear mixed models. Slight variations were tolerated knowing that confidence intervals of 95% were calculated through parametric bootstrap. The slope for the concentration was considered significantly different from zero at α = 0.05 if the 95% confidence interval did not contain zero. Full details and code for the analyses and to produce all figures are accessible at https://github.com/STATforU/sz_etoh_dmso.

## 5. Conclusions

Given the ubiquitous use of EtOH and DMSO as solvents for test molecules used to incubate spermatozoa, we believe that the results presented in the present study are of great relevance to the community of researchers working on sperm function.

Indeed, the intrinsic effect of EtOH and DMSO on sperm parameters must be taken into account when interpreting results obtained in the presence of the tested molecules, even if vehicle-treated controls are used. Based on the linear mixed models obtained to evaluate the impact of both solvents, we calculated the concentration of both solvents which would result in a 5% decline in sperm parameters. For EtOH, these concentrations are 0.9, 0.7, and 0.3% for total motility, progressive motility, and capacitation, respectively, while for DMSO they are 1.5, 1.1, >2, 0.3 and >2% for total motility, progressive motility, vitality, capacitation, and acrosome integrity, respectively (Table 4). We recommend using solvent concentrations below these values. Also, an untreated control group should always be included for all experiments in addition to the EtOH or DMSO vehicle control to evaluate the impact of the solvent on the investigated function or parameter.

## Figures and Tables

**Table 1:** Parameters of the semen samples included in the study, information from the donors, and the experiments for which they were used.

Donor	Age (years)	Volume (mL)	Viscosity	pH	Sperm Count (×10^6^ Sperm/mL)	Total Motility (%)	Progressive Motility (%)	Normal Morphology (%)	Experiment
1	30	2.8	normal	8.1	38.5	79.3	63.2	4	C, M, V
2	31	2.9	normal	7.9	114.07	56.1	30	5.79	C, M, V
3	29	6	normal	8.5	110.3	80.3	53.8	<1	C, M, V
4	21	6	increased	8.1	62.93	85.14	54.16	4	C, M, V
5	30	3	normal	8.1	51.5	70.9	45.7	4.1	C, M, V
6	25	2	increased	8.3	146.9	91.8	69.5	0.8	AI, M, MT
7	37	3	increased	8.1	121.2	88.7	67.7	0.81	AI, M, MT
8	30	3.9	increased	8.1	104.1	79.14	58.32	7.5	AI, M, MT
9	34	1.9	normal	7.9	80.78	74.5	59.9	1.7	AI, M, MT

Morphology values in red are outside the reference values provided by the World Health Organization (WHO) 2021 guidelines. AI: Acrosome integrity, C: capacitation, M: motility (concentration-effect), MT: motility (time effect), V: vitality.

**Table 2:** Influence of ethanol (EtOH) and dimethyl sulfoxide (DMSO) on sperm parameters, shown through fitting linear mixed models. The fixed effect coefficient for the concentration of solvents is shown with 95% confidence intervals and the number of donors, n.

Parameter	EtOH		DMSO	
	Coefficient (95% CI)	n	Coefficient (95% CI)	n
Motile sperm (%)	−0.30 [−0.39, −0.22] *	8	−0.19 [−0.28, −0.10] *	8
Progressively motile sperm (%)	−0.34 [−0.41, −0.26] *	8	−0.22 [−0.30, −0.13] *	8
Live sperm (%)	−0.032 [−0.16, 0.10]	5	−0.24 [−0.37, −0.11] *	5
Relative phosphotyrosine content	−0.15 [−0.28, −0.0048] *	5	−0.16 [−0.29, −0.021] *	5
Acrosome-intact sperm (%)	−0.15 [−0.32, 0.022]	4	−0.28 [−0.43, −0.12] *	4

Purified human spermatozoa were incubated for 4 h in a capacitation medium in the presence of 0, 0.1, 0.5, 1, and 2% EtOH or DMSO. For each parameter (except for phosphotyrosine content), a generalized linear mixed model, whose formula is (X, X_t_ − X) ~ Concentration + (Concentration | Donor), was fit and the fixed effect coefficient for concentration was reported. Parameters reported as percentages were logit transformed before fitting. Regarding capacitation, measured as relative densitometry phosphotyrosines/β-Tubulin, a linear mixed-effects model, whose formula is X_c_ ~ concentration + (1 | donor), was fit and the fixed effect coefficient for concentration was reported in the table. * indicate values significantly different from zero at α = 0.05 (i.e., the 95% interval confidence does not contain zero). X: number of motile, live, or acrosome-intact spermatozoa, X_t_: total number of counted spermatozoa according to the parameter of interest, X_c_: Relative Phosphotyrosine content.

**Table 3:** Influence of 1% and 2% ethanol (EtOH) and 2% dimethyl sulfoxide (DMSO) over time on sperm total motility, shown through fitting linear mixed models. The fixed effect coefficient for time (for the control condition) and for the difference of time (comparison between the tested and control conditions) are shown with 95% confidence intervals.

Parameter	Control	1% EtOH	2% EtOH	2% DMSO
	Time (95% CI)	Difference of Time (95% CI)	Difference of Time (95% CI)	Difference of Time (95% CI)
Motile sperm (%)	−0.062 [−0.15, 0.023]	0.11 [−0.0072, 0.23]	0.018 [−0.099, 0.14]	−0.075 [−0.19, 0.037]

Purified human spermatozoa were incubated at different incubation times (0, 0.5, 2, and 4 h) in a capacitation medium in the presence of 1% EtOH, 2% EtOH, or 2% DMSO. A generalized linear mixed model, whose formula is (X, X_t_ − X) ~ Treatment * Time + (1 | Donor), was fit and the fixed effect coefficient for time (slope of the model for the control condition without solvents) and for the difference of time (difference in slope, i.e., comparison between the tested and control conditions) were reported. Percentages of motility (n = 4) were logit transformed before fitting. No 95% interval confidence contains zero. X: number of motile spermatozoa, X_t_: total number of counted spermatozoa.

**Table 4:** Concentration of ethanol (EtOH) and dimethyl sulfoxide (DMSO) predicted to induce a 5% decline in sperm parameters using the linear mixed models.

Parameter	EtOH (%)	DMSO (%)
Motile sperm (%)	0.9	1.5
Progressively motile sperm (%)	0.7	1.1
Live sperm (%)	†	>2 *
Relative phosphotyrosine content	0.3	0.3
Acrosome-intact sperm (%)	†	>2 *

Values were calculated using equations available at https://github.com/STATforU/sz_etoh_dmso. Note that, here, predictions were made using mean values obtained for each parameter in the absence of solvent as the ordinate of origin, i.e., 0.8 for total motility, 0.7 for progressive motility, 0.94 for vitality and acrosome integrity, and 1.1 for relative phosphotyrosine content. * Effects of concentrations above 2% could not be predicted by the linear models as they were fitted using data obtained for concentrations ranging between 0 and 2%. † indicate a statistically non-significant impact of the solvent.

## Data Availability

The data presented in this study are available in Supplementary Materials (see above) and in https://github.com/STATforU/sz_etoh_dmso.

## Supplementary Materials

The supporting information can be downloaded at: https://www.mdpi.com/article/10.3390/ijms24010505/s1.
