# Supplementary material for: Influence of Two Widely Used Solvents, Ethanol and Dimethyl Sulfoxide, on Human Sperm Parameters
Source: Int J Mol Sci. 2022 Dec 28;24(1):505. doi: 10.3390/ijms24010505 (PMC9820180; doi:10.3390/ijms24010505)
Supplement: Supplementary file 1 [file ijms-24-00505-s001.zip › ijms-2094910-supplementary.pdf]

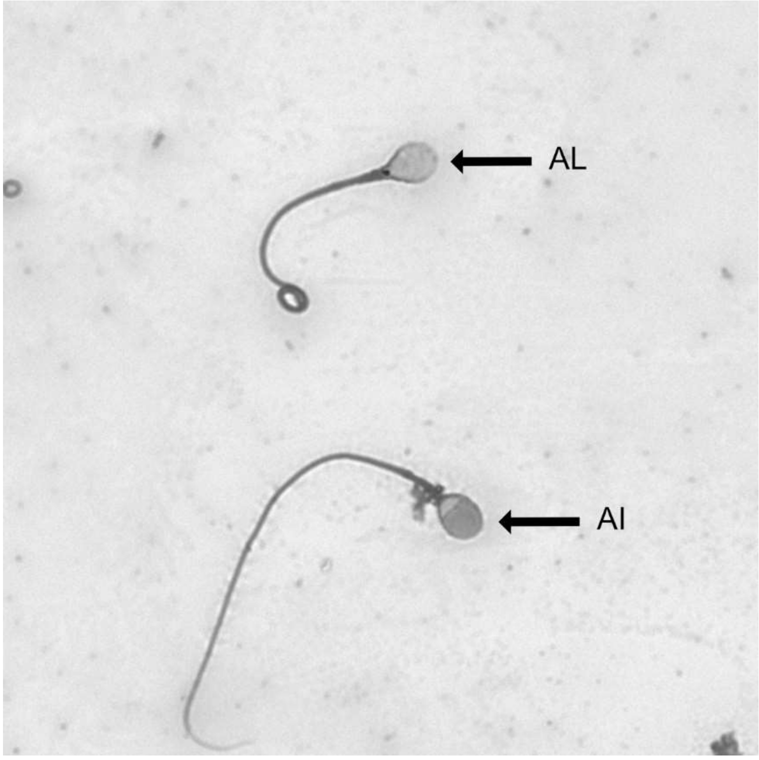

Figure S1. Sperm acrosome stained with Coomassie Blue G-250. After incubation for 4 h in a capacitation medium, spermatozoa were fixed in 4% paraformaldehyde in PBS, washed, air-dried on a microscope slide and stained with a Coomassie stain (0.22% Coomassie Blue G-250, 50% methanol, 10% acetic acid and 40% water) for 2 min. AI: acrosome-intact, AL: acrosome-less.

Table S1. Raw data for the influence of EtOH and DMSO on sperm parameters → Excel file.

Table S2. Raw data for the influence of 1% and 2% EtOH and 2% DMSO on sperm motilities over time → Excel file.

Table S3. Influence of ethanol (EtOH) and dimethyl sulfoxide (DMSO) on sperm parameters, shown through fitting linear mixed models. The fixed effect coefficient for intercept and concentration of solvents is shown with 95% confidence intervals and number of donors n.

**Parameter**	**EtOH**			**DMSO**		
	Intercept (95% CI)	Concentration (95% CI)	n	Intercept (95% CI)	Concentration (95% CI)	n
**Motile sperm (%)**	1.27 [0.97, 1.58]	-0.30 [-0.39, -0.22]*	8	1.40 [1.15, 1.67]	-0.19 [-0.28, -0.10]*	8
**Progressively motile sperm (%)**	0.80 [0.48, 1.12]	-0.34 [-0.41, -0.26]*	8	0.91 [0.68, 1.16]	-0.22 [-0.30, -0.13]*	8
**Live sperm (%)**	2.77 [2.49, 3.08]	-0.032 [-0.16, 0.10]	5	2.91 [2.65, 3.22]	-0.24 [-0.37, -0.11]*	5
**Relative phosphotyrosines content**	1.14 [0.99, 1.29]	-0.15 [-0.28, -0.0048]*	5	1.04 [0.88, 1.22]	-0.16 [-0.29, 0.021]*	5
**Acrosome-intact sperm (%)**	2.66 [2.40, 2.96]	-0.15 [-0.32, 0.022]	4	2.71 [2.44, 2.99]	-0.28 [-0.43, -0.12]*	4

Purified human spermatozoa were incubated for 4 h in a capacitation medium in the presence of 0, 0.1, 0.5, 1 and 2% EtOH or DMSO. For each parameter (excepted for phosphotyrosine content), a generalized linear mixed model, whose formula is $(X, X_t - X) \sim$ Concentration + (Concentration | Donor), was fit and the fixed effect coefficient for concentration reported. Parameters reported as percentages were logit transformed before fitting. Regarding capacitation, measured as relative densitometry phosphotyrosines/β-Tubulin, a linear mixed-effects model, whose formula is $X_c \sim$ concentration + (1 | donor), was fit and the fixed effect coefficient for concentration reported in the table. *indicate values significantly different from zero at $\alpha = 0.05$ (i.e., the 95% interval confidence do not contain zero). X: number of motile, live or acrosome intact spermatozoa, X_t : total number of spermatozoa counted according to the parameter of interest, X_c : Relative Phosphotyrosine content.

Table S4. Influence of 1% and 2% ethanol (EtOH) and 2% dimethyl sulfoxide (DMSO) on total sperm motility, shown through fitting a generalized linear mixed model. The fixed effect coefficient for concentration of solvents is shown with 95% confidence intervals and number of donors n.

**Parameter**	**Control**		**1% EtOH**		**2% EtOH**		**2% DMSO**	
	Intercept (95% CI)	Time (95% CI)	Difference of intercept (95% CI)	Difference of time (95% CI)	Difference of intercept (95% CI)	Difference of time (95% CI)	Difference of intercept (95% CI)	Difference of time (95% CI)
**Motile sperm (%)**	1.82 [1.59, 2.07]	-0.062 [-0.15, 0.023]	-0.52 [-0.82, -0.25]	0.11 [-0.0072, 0.23]	-0.57 [-0.85, 0.31]	0.018 [-0.099, 0.14]	0.079 [-0.20, 0.36]	-0.075 [-0.19, 0.037]

Purified human spermatozoa were incubated at different incubation times (0, 0.5, 2 and 4 h) in a capacitation medium in the presence of 1% EtOH, 2% EtOH or 2% DMSO. A generalized linear mixed model, whose formula is $(X, X_t - X) \sim \text{Treatment} \times \text{Time} + (1 \mid \text{Donor})$, was fit and the fixed effect coefficient for time reported. Percentages of motile spermatozoa (n = 4) were logit transformed before fitting. No 95% confidence interval contains zero. X: number of motile spermatozoa, X_t : total number of counted spermatozoa.
